# Axial Length Shortening after Combined Repeated Low-Level Red-Light Therapy in Poor Responders of Orthokeratology in Myopic Children

**DOI:** 10.1155/2024/4133686

**Published:** 2024-08-10

**Authors:** Mengting Yu, Xianghua Tang, Jinyun Jiang, Fengqi Zhou, Lili Wang, Chuqi Xiang, Yin Hu, Xiao Yang

**Affiliations:** ^1^ State Key Laboratory of Ophthalmology Zhongshan Ophthalmic Center Sun Yat-sen University, Guangzhou, China; ^2^ Mayo Clinic College of Medicine and Science, Rochester, Minnesota, USA; ^3^ Department of Ophthalmology Mayo Clinic Health System, Eau Claire, Wisconsin, USA

## Abstract

**Purpose:**

To investigate the efficacy and safety of orthokeratology (ortho-k) and repeated low-level red-light (RLRL) therapy in treating poor responders of ortho-k in myopic children.

**Methods:**

Study participants were 100 myopic children who completed two years of ortho-k treatment in a retrospective study. In the first year of ortho-k treatment (phase one), they experienced axial elongation of 0.30 mm or greater (defined as poor responders to ortho-k). Children were divided into two groups: the orthokeratology group (OK, *n* = 45) continued to receive ortho-k monotherapy and the combination group (OK-RLRL, *n* = 55) received RLRL in addition to ortho-k for the next year (phase two). Axial elongation over time between the groups was compared.

**Results:**

The mean age, male-to-female ratio, axial length (AL), and axial elongation in phase one were comparable between OK and OK-RLRL groups (all *P* > 0.05). During phase two, significant AL shortening was observed in the OK-RLRL group compared with children in the OK group (−0.10 ± 0.16 mm vs 0.30 ± 0.19 mm, *P* < 0.001). Among these 55 myopic children in the OK-RLRL group, 35 (63.6%), 25 (45.4%), 11 (20%), 6 (10.9%), and 3 (5.4%) of them had AL shortening over 0.05 mm/year, 0.10 mm/year, and 0.20 mm/year, 0.3 mm/year, and 0.4 mm/year, respectively. Older baseline age (*β* = −0.02), higher treatment compliance (*β* = −0.462), and AL change at 1 month (*β* = 1.263) were significantly associated with less AL elongation (all *P* < 0.05).

**Conclusions:**

For poor responders of orthokeratology, RLRL could slow axial elongation in addition to the ortho-k treatment effect. Those who respond poorly to ortho-k with elder age might benefit more from combined therapy.

## 1. Introduction

Myopia is one of the most common ocular diseases globally, characterized primarily by excessive axial elongation of the eye [1]. The prevalence of juvenile myopia has reached an alarming level around the world, especially in East and Southeast Asia [1–3]. Myopia progression typically begins in the elementary school years and progresses in the second decade of life. Excessive axial elongation places individuals at a higher risk of pathological changes in later life, including cataract, glaucoma, macular diseases, posterior staphyloma, and chorioretinal degenerations. Therefore, it is crucial to implement measures to slow down myopia progression in children.

Many interventions have been proposed to slow down the myopia progression [4], including pharmacological therapies, optical treatments, and behavioral modifications. Among these myopia control modalities, orthokeratology (ortho-k), which uses specially designed, reverse geometry, gas-permeable contact lenses to temporarily correct myopia by reshaping the cornea, has gained popularity worldwide. Though accumulating studies have shown that ortho-k could reduce myopia progression [5], the efficacy of ortho-k on slowing down the progression of axial elongation varied from individuals, with a mean control rate of 30–59% [6–8]. As indicated by previous research studies, many influencing factors have been proposed accounting for this individual variation [9, 10], involving the patient's age of myopia onset, initial refractive error, degree and progression rate of myopia, and pupil size. Therefore, methods are needed to better assist those who respond poorly to ortho-k, defined as experiencing over 0.3 mm axial elongation within one year of wearing ortho-k lenses [11], to effectively slow down myopia progression and axial elongation.

Repeated low-level red-light therapy (RLRL), an emerging treatment for myopia control, which delivers red light on the retina directly at a much shorter duration of exposure, has shown promising results in myopia prevention [12] and control [13–15]. A published multicenter randomized clinical trial study [14] has reported RLRL treatment significantly retarded axial elongation and spherical equivalent refraction progression over 12 months compared with single vision spectacles, representing a 69.4% and 76.6% slowing axial elongation and myopic refraction progression. Therefore, this preliminary study was attempted to investigate the efficacy and safety of ortho-k and RLRL on myopia control in poor responders of ortho-k with at least 12 months. Herein, we reported the safety and effect of the combination therapy for 12 months.

## 2. Methods

### 2.1. Study Design

Chinese children who visited Zhongshan Ophthalmic Center, Sun Yat-sen University, between January 2020 and February 2022 and who met the following criteria were enrolled in this retrospective study: (1) aged between 7 and 15 years; (2) ortho-k poor responders with axial elongation ≥0.30 mm during one year of ortho-k treatment [11]. When both eyes were eligible, the right eye was selected.

All participants received comprehensive examination before being fitted with either spherical CRT (Paragon Vision Sciences, Mesa, AZ, United States) or four-zone ortho-k lenses (Euclid Systems Orthokeratology, Euclid System, Herndon, VA, USA; DreamLite, Procornea Nederland B.V., Gelderland, Netherlands) in both eyes. A trial lens fitting strategy has been applied in all cases, and lenses were ordered targeting full correction. Follow-up clinic visits were scheduled at 1 day, 1 week, 1 month, 3 months, 6 months, and every 6 months after ortho-k lens wear. Participants were advised to wear their lenses every night for at least seven consecutive hours unless otherwise instructed. Ortho-k lens prescription was modified only when an unaided visual acuity of 20/25 was not achieved or significant lens decentration was observed. Ocular axial length (AL) was measured in both eyes at baseline, 1 month, 6 months, and 12 months after commencing ortho-k lens wear. Participants who showed axial elongation of 0.3 mm or greater during one year of ortho-k were advised to receive repeated low-level red-light therapy or low-concentration atropine in addition to ortho-k treatment. Those who chose to combine RLRL were included in the combination group (orthokeratology and repeated low-level red-light therapy, OK-RLRL group), and the rest of ortho-k poor responders whose parents considered to continue ortho-k monotherapy were included as the control group (orthokeratology, OK group). The study and data acquisition were carried out with approval from the Ethical Committee of Zhongshan Ophthalmic Center, Sun Yat-sen University (2022KYPJ100), and adhered to the tenets of the Declaration of Helsinki.

Participants in the OK-RLRL group were instructed to receive low-level, 650 nm laser therapy (Eyerising, Suzhou Xuanjia Optoelectronics Technology, China) twice a day, for 3 minutes each session, with a minimum interval of 4 hours between sessions. The device is certified as a class IIa device by the China National Medical Products Administration [12–14], which is equipped with semiconductor laser diodes which emits 650 ± 10 nm red light, with a laser power of 0.29 mW through a 4 mm pupil [13, 14]. Intervention compliance was monitored through an automated diary function built into the system. The device, connected to the Internet, recorded the exact dates and times of the treatment sessions, providing an accurate measure of treatment compliance. Compliance was calculated as the percentage of actual completed sessions relative to the scheduled treatment sessions (twice daily, seven days a week) over the entire treatment period.

All participants were evaluated every 6 months and underwent a comprehensive ophthalmologic examination, including slit-lamp microscopy, refraction, corneal topography, measurement of unaided visual acuity, and best corrected visual acuity (BCVA). Ocular axial length was measured in both eyes using the noncontact optical biometry device (Iolmaster500, Carl Zeiss Meditec, Germany), with average of five repeated measurements used for analysis. Examinations were performed in the morning to minimize effects of circadian variation. Participants in the OK-RLRL group were followed up after one month of combination of RLRL combination, accessing visual acuity, AL, and possible discomfort symptoms involving glare, flash blindness, and afterimage. At 12 months of follow-up, fundus imaging examination was taken to monitor the occurrence of adverse events, including a scan of the macular area obtained by optical coherence tomography (VG200; SVision Imaging, Ltd., Luoyang, China) and wide-angle color fundus photography performed by Daytona Optos device (Optos, Dunfermline, UK).

### 2.2. Statistical Analysis

Descriptive statistics were applied. Means ± standard deviations, medians (interquartile ranges, IQR), and numbers and percentages were reported where appropriate. The significance of between-group differences was determined using *t* test if the data were normally and equally distributed. Axial elongation rates during phase one and phase two were compared using the paired *t* test. Categorical variables were presented as counts and percentages and compared with the chi-square test when appropriate (expected frequency >5). Multiple linear regression models assessed baseline factors associated with changes in axial length in the OK-RLRL group over 12 months. Potential covariates included baseline age, gender, AL, treatment compliance, and AL change at one month after the combination of RLRL. Factors with *P* values <0.10 in univariate models were entered into the multivariate models. AL shortening was defined as AL reduction over 0.05 mm from the baseline.

All statistical analyses were performed using SPSS (version 24, SPSS, Inc.). A *P* value <0.05 was considered statistically significant.

## 3. Results

A total of 100 eyes from 100 participants (45 OK, 55 OK-RLRL) were included in this study. The distribution of baseline age (10.59 ± 1.47 years vs 10.66 ± 1.86 years, *P*=0.822), gender, and axial length (25.48 ± 1.10 mm vs 25.73 ± 1.22 mm, *P*=0.28) were balanced between OK and OK-RLRL groups, respectively.

**Figure 1:**
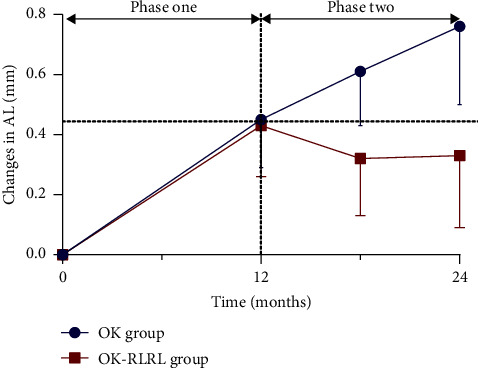
Axial length change during phase one and phase two in two groups. OK group, orthokeratology group; OK-RLRL, orthokeratology combined with repeated low-level red light.

In phase one, there was no significant difference in axial elongation (0.45 ± 0.16 vs 0.43 ± 0.17 mm, *P*=0.53) between OK and OK-RLRL groups, respectively. During phase two, AL elongation in the OK-RLRL group was significantly lower than that in the OK group (−0.10 ± 0.16 mm vs 0.30 ± 0.19 mm, *P* < 0.001) (Figure 1). After 1 month of combining RLRL, there was a significant axial shortening of −0.05 ± 0.06 mm, with further reductions observed at 6 months and 1 year of combined treatment in the OK-RLRL group.

**Figure 2:**
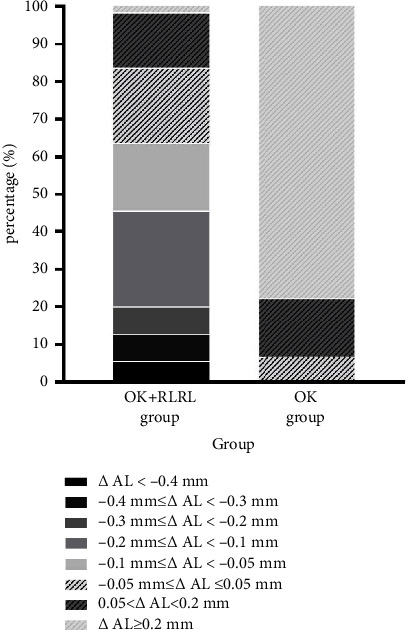
Distribution of axial length change during phase two in two groups ΔAL, annual change in axial length, positive value represents axial elongation and negative value represents axial shortening; OK group, orthokeratology group; OK-RLRL, orthokeratology combined with repeated low-level red light.

Among these 55 myopic children in the OK-RLRL group, 35 (63.6%), 25 (45.4%), 11 (20%), 6 (10.9%), and 3 (5.4%) of them had AL shortening over 0.05 mm/year, 0.10 mm/year, 0.20 m/year, 0.3 mm/year, and 0.4 mm/year, respectively (Figure 2).

Compliance of RLRL treatment throughout the study was excellent as 73% of the patients reached the minimum compliance of 80%. The median (IQR) treatment compliance was 90% (75%, 95%) (range, 54%–99%) in the OK-RLRL group. In the multivariable model, older baseline age, higher treatment compliance, and AL change at 1 month were significantly associated with less AL elongation (Table 1).

All participants underwent uneventful ortho-k treatment in both phases. In the OK-RLRL group, none reported any glare or flash blindness following the addition of RLRL. At the final follow-up, wide-angle color fundus photography revealed no significant retinal degeneration or structural retinal vascular changes. Similarly, OCT scans showed no structural damage in the photosensory layer. The application of RLRL was generally well tolerated, and no systemic adverse events were reported.

## 4. Discussion

In the present study, we investigated the safety and efficacy of ortho-k in combination with RLRL for the control of progressive axial elongation in those who responded poorly to ortho-k, and the preliminary results were encouraging. All the enrolled subjects were originally identified as “poor responders of orthokeratology”. In phase one, mean axial elongation was 0.45 ± 0.16 mm/year and 0.43 ± 0.17 mm/year in both groups, which were faster than that reported in previous studies [6, 16]. In phase two, axial elongation continued in the ortho-k monotherapy group, while a significant axial shortening was observed after 12 months of combination with RLRL in the combination group (0.30 ± 0.19 mm vs −0.10 ± 0.16 mm, *P* < 0.001). In the OK-RLRL group, baseline age, treatment compliance, and AL change at 1 month that were found correlated significantly with long-term change in AL at 12 months. Apart from being effective, RLRL was well tolerated generally and no serious adverse effects were observed.

For those who responded unsatisfactory to ortho-k in terms of axial length control, limited effective treatments have been reported. Chen et al. [11, 17] attempted to add low-level atropine to ortho-k (OKA), showing combining 0.01% nightly atropine with ortho-k slowed axial elongation in the following year. However, when the authors extended the combination therapy to 2 years, the OKA group revealed no consistent superiority in myopia control over the OK group in the second year (0.23 ± 0.13 mm vs 0.20 ± 0.13 mm) [11]. Our preliminary study [18] indicated that RLRL had potential benefits for children who were poor responders to orthokeratology. In the present study, when RLRL was added, a dramatic axial shortening was found in the following 12 months, with a mean AL reduction of 0.10 ± 0.16 mm. A more thorough evaluation, including assessments of ocular biometrics, and retinal and choroidal vessel density, is crucial to gain a deeper understanding of the key factors that influence the axial shortening process. Furthermore, extended research is necessary to monitor the long-term efficacy and safety of combining ortho-k with red light therapy.

Axial shortening in myopia has been reported in limited clinical studies, though it is generally assumed that axial elongation is irreversible. On the pharmaceutic arm, ATOM1 study has demonstrated that 1% ATP could effectively suppress axial elongation by −0.14 ± 0.28 mm in the first year [19]. In addition, in the LAMP study aiming at lower dose of atropine, approximately 6% of children who adopted 0.05% atropine eye drops had an axial shortening of 0–0.25 mm during the two-year follow-up [20]. The possible mechanism of atropine in reducing axial growth might be that atropine acts on muscarinic receptor in the retina and/or sclera causing inhibition of vitreous chamber elongation and choroidal thinning [21]. On the optical arm, using the same device emitting repeated low-level red light (650 nm, 1600 lx), Jiang et al. [14] reported AL shortening over 0.05 mm in 39.8% of the participants at 1 month and in 21.6% of the participants at 12 months in RLRL group in a multicenter randomized clinical trial. Further analysis showed that the amount of axial shortening could only be partially explained by thickened choroid (40 *μ*m vs 16.1 *μ*m). In addition, a real-world retrospective study demonstrated 26.50% of myopic children underwent AL shortening over 0.05 mm following over 12 months of RLRL therapy [21]. Similarly, in our study, clinically significant AL shortening over 0.05 mm was also observed in 65.45% (36/55) and 63.63% (35/55) of participants following 6 months and 12 months of combined strategy. The variation in axial shortening between studies may be attributed to differences in participant demographics. It appeared that elder age was significantly related to slower axial elongation after combination therapy, which was consistent with previous published studies regarding RLRL [20, 21]. Besides, it is important to note that our study focused on participants with progressive myopia, whereas other RLRL studies did not specify the rate of myopia progression [13, 14, 20]. Furthermore, it has been hypothesized that ortho-k induces relative peripheral myopic defocus on the retina by altering corneal peripheral refraction, thereby reducing the stimulus for eye elongation. The combined impact of these two treatments could potentially lead to a more substantial reduction in axial length. Despite the significant effect on slowing down or even shortening of axial elongation after the combination of RLRL, existing literature [15] has pointed out the possibility of rebound effect upon cessation of RLRL. Therefore, further research should track patients over longer periods to evaluate the rebound effect following the discontinuation of RLRL combined with ortho-k, as well as to identify the factors influencing the rebound effect. Light, as a crucial visual stimulus, is intimately linked to refractive development and ocular growth. Prior research has delved into the relationship between light signaling and myopia development, revealing that both the wavelength and intensity of light can influence myopia. Recently, red light has come into focus for myopia control. According to the hypothesis of longitudinal chromatic aberrations, red light should induce hyperopic defocus and thus counteract myopia. However, the impact of red light on myopia appears to vary across species. Myopia shift was found in chicks [22] and guinea pigs [23] rearing under 628 ± 10 nm and 600 ± 5 nm light environment. On the contrary, the 630 nm light used by Hung et al. [24] and the 624 ± 10 nm light used by Gawne et al. [25] showed significant effects on myopia control in rhesus monkeys and tree shrews. In the current study, 650 nm repeated low-level red light demonstrated the potential to slow myopia development and even reduced the axial length in those who responded poorly to ortho-k. The exact mechanism of RLRL in slowing myopia progression has not been clearly elucidated. It was speculated that the possible targets of RLRL on slowing myopia might be the choroid or sclera. Clinically, choroid thickening has been documented following RLRL, yet this alone does not fully account for the observed AL reduction [14]. Experimentally, 660 nm RLRL was demonstrated to promote the synthesis of extracellular matrix components, such as collagen I a1 (COL1A1), and improve hypoxia by regulating the expression of HIF-1*α* in human scleral fibroblasts in vitro in a hypoxic environment [26]. Besides, accumulating evidence has indicated that oxidative stress and inflammation may be involved in myopia, and oxidative damage associated with hypoxic myopia could reduce the neuromodulation of nitric oxide and dopamine during eye growth [27]. While RLRL has been proved to have effects on the nitric oxide system and decreases the severity of oxidative stress, enhance mitochondrial function and ATP synthesis [28] in previous studies, it is speculated that RLRL treatment might increase the release of nitric oxide to enhance vasodilation of the fundus and thus ameliorating scleral hypoxia. More experimental research is required to fully elucidate the specific mechanism of RLRL in inhibiting myopia progression.

The strength of this study was its high intervention fidelity, evidenced by an overall mean treatment adherence of 85%. There were several limitations in this study. First, the study design was retrospective in nature with a relative short follow-up time and therefore subject to selection bias. However, this work offered valuable insights into a potential strategy to those who responded poorly to ortho-k. We would further extend the follow-up duration to observe the myopia control effects between the two groups. Another limitation of the current study did not provide the corresponding ocular biometric data such as central corneal thickness, anterior chamber depth, lens thickness, vitreous chamber depth, and choroidal thickness. Additionally, the safety assessment in this study was based only on structural evaluations using OCT and SLO, without functional examinations such as microperimetry and contrast sensitivity. Studies evaluating these details could shed more light on the underlying mechanisms of this phenomenon.

In conclusion, this research indicated the potential combination of RLRL in children with rapidly progressing myopia with ortho-k and no documented functional or structural damage. Further studies are needed to determine the long-term efficacy and safety in clinical practice.

## Figures and Tables

**Table 1:** Linear regression analyses of the associations of the 12-month axial length change with baseline characteristics and the 1-month AL change in the OK-RLRL group.

	Univariate model		Multivariate model	
	*β* (95% CI)	*P* value	*β* (95% CI)	*P* value
Age (years)	−0.026 (−0.049, −0.003)	0.026	−0.02 (−0.004, −0.01)	0.04
Gender (boys)	−0.022 (−0.112, 0.069)	0.632	—	—
Baseline AL (mm)	−0.018 (−0.055, 0.02)	0.35	—	—
AL change in phase one (mm)	0.056 (−0.202, 0.314)	0.666	—	—
1-month AL change	1.431 (0.719, 2.142)	<0.001	1.263 (0.6, 1.925)	<0.001
Treatment compliance	−0.438 (−0.777, −0.099)	0.012	−0.462 (−0.749, −0.175)	0.002

AL, axial length.

## Data Availability

The datasets generated during and/or analyzed during the current study are available from the corresponding author on reasonable request.

## References

[1] Resnikoff S., Jonas J. B., Friedman D. (2019). Myopia—a 21st century public health issue. *Investigative Ophthalmology and Visual Science*.

[2] Holden B. A., Fricke T. R., Wilson D. A. (2016). Global prevalence of myopia and high myopia and temporal trends from 2000 through 2050. *Ophthalmology*.

[3] Sankaridurg P., Tahhan N., Kandel H. (2021). IMI impact of myopia. *Investigative Ophthalmology and Visual Science*.

[4] Jonas J. B., Ang M., Cho P. (2021). IMI prevention of myopia and its progression. *Investigative Ophthalmology and Visual Science*.

[5] Zhong Y., Chen Z., Xue F., Zhou J., Niu L., Zhou X. (2014). Corneal power change is predictive of myopia progression in orthokeratology. *Optometry and Vision Science*.

[6] Cho P., Cheung S. W. (2012). Retardation of myopia in orthokeratology (ROMIO) study: a 2-year randomized clinical trial. *Investigative Ophthalmology and Visual Science*.

[7] Kakita T., Hiraoka T., Oshika T. (2011). Influence of overnight orthokeratology on axial elongation in childhood myopia. *Investigative Ophthalmology and Visual Science*.

[8] Swarbrick H. A., Alharbi A., Watt K., Lum E., Kang P. (2015). Myopia control during orthokeratology lens wear in children using a novel study design. *Ophthalmology*.

[9] Chen Z., Niu L., Xue F. (2012). Impact of pupil diameter on axial growth in orthokeratology. *Optometry and Vision Science*.

[10] Santodomingo-Rubido J., Villa-Collar C., Gilmartin B., Gutiérrez-Ortega R. (2013). Factors preventing myopia progression with orthokeratology correction. *Optometry and Vision Science*.

[11] Chen Z., Zhou J., Xue F., Qu X., Zhou X. (2022). Two-year add-on effect of using low concentration atropine in poor responders of orthokeratology in myopic children. *British Journal of Ophthalmology*.

[12] He X., Wang J., Zhu Z. (2023). Effect of repeated low-level red light on myopia prevention among children in China with premyopia: a randomized clinical trial. *JAMA Network Open*.

[13] Dong J., Zhu Z., Xu H., He M. (2023). Myopia control effect of repeated low-level red-light therapy in Chinese children. *Ophthalmology*.

[14] Jiang Y., Zhu Z., Tan X. (2022). Effect of repeated low-level red-light therapy for myopia control in children. *Ophthalmology*.

[15] Xiong R., Zhu Z., Jiang Y. (2022). Sustained and rebound effect of repeated low-level red-light therapy on myopia control: a 2-year post-trial follow-up study. *Clinical and Experimental Ophthalmology*.

[16] Zhong Y., Chen Z., Xue F., Miao H., Zhou X. (2015). Central and peripheral corneal power change in myopic orthokeratology and its relationship with 2-year axial length change. *Investigative Ophthalmology and Visual Science*.

[17] Chen Z., Huang S., Zhou J., Xiaomei Q., Zhou X., Xue F. (2019). Adjunctive effect of orthokeratology and low dose atropine on axial elongation in fast-progressing myopic children—a preliminary retrospective study. *Contact Lens and Anterior Eye*.

[18] Yu M., Yang X., Hu Y., Li Z. International myopia conference abstract book. https://www.internationalmyopiaconference.org/wp-content/uploads/2022/09/IMC-2022-Abstract-book.pdf.

[19] Chua W. H., Balakrishnan V., Chan Y. H. (2006). Atropine for the treatment of childhood myopia. *Ophthalmology*.

[20] Li F. F., Zhang Y. Z., Zhang X. J. (2021). Age effect on treatment responses to 0.05%, 0.025%, and 0.01% atropine: low-concentration atropine for myopia progression study. *Ophthalmology*.

[21] Nickla D. L., Zhu X., Wallman J. (2013). Effects of muscarinic agents on chick choroids in intact eyes and eyecups: evidence for a muscarinic mechanism in choroidal thinning. *Ophthalmic and Physiological Optics*.

[22] Wang W., Jiang Y., Zhu Z. (2023). Clinically significant axial shortening in myopic children after repeated low-level red light therapy: a retrospective multicenter analysis. *Ophthalmology and Therapy*.

[23] Jiang L., Zhang S., Schaeffel F. (2014). Interactions of chromatic and lens-induced defocus during visual control of eye growth in Guinea pigs (Cavia porcellus). *Vision Research*.

[24] Hung L. F., Arumugam B., She Z., Ostrin L., Smith E. L. (2018). Narrow-band, long-wavelength lighting promotes hyperopia and retards vision-induced myopia in infant rhesus monkeys. *Experimental Eye Research*.

[25] Gawne T. J., Ward A. H., Norton T. T. (2017). Long-wavelength (red) light produces hyperopia in juvenile and adolescent tree shrews. *Vision Research*.

[26] Zhang P., Zhang X., Zhu H. (2023). Photobiomodulation at 660 nm promotes collagen synthesis via downregulation of HIF-1*α* expression without photodamage in human scleral fibroblasts in vitro in a hypoxic environment. *Graefes Archive for Clinical and Experimental Ophthalmology*.

[27] Francisco B. M., Salvador M., Amparo N. (2015). Oxidative stress in myopia. *Oxidative Medicine and Cellular Longevity*.

[28] Quirk B. J., Whelan H. T. (2020). What Lies at the heart of photobiomodulation: light, cytochrome C oxidase, and nitric oxide-review of the evidence. *Photobiomodulation, Photomedicine, and Laser Surgery*.

